# Immune-Activated Regional Lymph Nodes Predict Favorable Survival in Early-Stage Triple-Negative Breast Cancer

**DOI:** 10.3389/fonc.2020.570981

**Published:** 2020-10-09

**Authors:** Yi-Yu Chen, Jing-Yu Ge, Ding Ma, Ke-Da Yu

**Affiliations:** Department of Breast Surgery, Shanghai Medical College, Shanghai Cancer Center and Cancer Institute, Fudan University, Shanghai, China

**Keywords:** triple-negative breast cancer, lymph node, immune response, survival, immune activation

## Abstract

Immune response and immunotherapy play important roles in triple-negative breast cancer (TNBC). However, it is difficult to judge whether cancer is “immune-inactivated” or “immune-activated” by the carcinoma itself. The immune reaction of the microenvironment or the host to the tumor might be more informative. We assumed that clinically enlarged but pathologically negative regional lymph nodes served as an indicator for early immune response to tumors. First, we identified women with pN0 breast cancer disease from the current Surveillance, Epidemiology, and End Results database, and we compared the cN1 patients of breast cancer-specific survival (BCSS) with cN0 patients. Then, we extracted total RNA from 36 paired large (defined as minimum diameter more than 15 mm in size) and small lymph nodes (defined as maximum diameter less than 5 mm in size) from 12 TNBC, 12 HER2-enriched, and 12 luminal-like patients and performed RNA sequencing to explore the gene expression and cellular landscape of large nodes compared to small ones. Among 692 women with pathologically confirmed node-negative disease, cN1 patients unexpectedly had a better BCSS compared with cN0 in TNBC (adjusted hazard ratio 0.148, 95% CI, 0.040–0.546, *P* = 0.004) but not in other subtypes. Further transcriptome sequencing of 12 paired enlarged and small negative nodes from TNBC patients revealed that increased immune activation signaling (e.g., interferon-gamma response pathways) and abundant immune cells (activated dendritic cells, CD4+ and CD8+ T-cells) were more frequently observed in enlarged nodes. Our data implied that early immune activation in regional lymph nodes in TNBC might affect survival.

## Introduction

Among females, breast cancer is the most commonly diagnosed cancer and the leading cause of cancer death, followed by colorectal and lung cancer for incidence, and vice versa for mortality (1). Considering its heterogeneous biological nature, breast cancer can be clinically stratified into three main subtypes: luminal-like, human epidermal growth factor receptor 2 (HER2)-enriched, and triple-negative breast cancer (TNBC), according to the status of three critical receptors: estrogen receptor (ER), progestogen receptor (PR), and HER2 (2). That molecular information, in coordination with clinical pathological information, was used to predict the outcomes of patients and helped to make the therapeutic decisions (3).

Immunotherapy in combination with chemotherapy has shown promising efficacy across many different tumor types (4). The treatment of several kinds of malignancies with immune checkpoint inhibitors (against programmed death receptor-1/ligand-1 [PD-1/PD-L1]) has changed the treatment panorama (5, 6). In TNBC, which is a difficult-to-treat disease with a high unmet therapeutic need, the IMpassion130 clinical trial has recently granted an accelerated approval for atezolizumab, an antibody targeting PD-L1, for patients with PD-L1-positive advanced TNBC (7). Judging a breast carcinoma as immune-reactive or immune-unreactive is still difficult. For instance, in IMpassion130, PD-L1-positive status was defined as PD-L1 expression on tumor infiltrating immune cells of 1% or more, indicating that it is important to take the cancer stroma or microenvironment into consideration (7). In other words, it is difficult to judge “immune-inactivated” or “immune-activated” by the carcinoma itself; the reactions of the microenvironment or host to the tumor might be more informative.

Regional lymph nodes, which provide the clues for initial tumor metastasis, are among the most important prognostic determinants. There are two main types of evaluation for regional lymph node status: clinical and pathological. The clinical assessment gives the estimation of lymph nodes preoperatively according to the physical examination and imaging modality and, thus, is crucial for the following surgical decision-making. Pathological evaluation, based on the findings during or after surgery with detailed pathological information, gives the most precise assessment of lymph nodes to direct adjuvant treatment and the prediction of survival outcomes. Sometimes there is inconsistency between these two types of estimates, usually in the cases in which clinical assessments underestimate the extent of the disease (8). However, there is another segment of the population whose negative pathological results of lymph nodes go against the positive clinical ones (9), where much uncertainty still exists about their clinicopathological features and prognosis. Considering that regional lymph nodes are parts of the host’s immune system, we hypothesized that clinically enlarged but pathologically negative regional lymph nodes might serve as an indicator for early systemic immune response to tumor and that immune activation probably resulted in an improved survival outcome of breast cancer patients. To test this hypothesis, we conducted the present study.

## Materials and Methods

### Study Population

The current Surveillance, Epidemiology, and End Results (SEER) database consists of 18 population-based tumor registries, covering approximately 34.6% of the United States population. The SEER program collects data on patients’ demographics, tumor characteristics, the first course of treatment, and survival outcomes. SEER^∗^stats 8.3.5 and Nov 2017 submission with the years of diagnosis varying from 2010 to 2015 was used to generate the patient list.

Since the adjusted American Joint Committee on Cancer (AJCC) lymph node categories for breast cancer in the SEER database did not separate clinical from pathologic information, we mainly used the code “CS Regional Node Evaluation” (coding 0,1,5,9), which derived the staging basis (clinically or pathologically) for lymph node category, to extract patients with clinical lymph node (cN) information. Because of the absence of data on HER2 status of patients diagnosed before 2010, we identified eligible patients according to the following criteria: diagnosed between 2010 and 2015, female, aged between 18 and 70 years, breast cancer as the first cancer diagnosis, microscopically confirmed infiltrating ductal carcinoma, unilateral, pT1-T2, cN0-N1, surgery performed, and regional lymph node examined to be pathologically negative (pN0). Patients with cN2 status were excluded, as they might be receiving neoadjuvant chemotherapy. Although it is the best method to establish clinical node stage using fine-needle aspiration, in clinical routine, not all the patients would undergo fine-needle aspiration and the SEER databased did not provide such information. To ensure the accuracy of pathological lymph node assessment, the number of lymph nodes dissected in therapeutic surgery was at least 10 for each patient. Subsequently, patients with unknown data on race, tumor grade, ER and PR status, as well as HER2 status, were excluded. As a result, we identified 692 breast cancer patients who satisfied our research purpose from the SEER database. Eligible patients were classified as luminal-like (ER and/or PR-positive, any HER2 status), HER2-enriched (ER and PR-negative, HER2-positive), and TNBC (ER, PR, and HER2-negative) subgroups.

### Enlarged and Small Lymph Node Samples

The preferred cutoff of size should be 10 mm for cN1 and cN0, because nodes are generally considered to be normal if they are less than 10 mm in diameter. However, some investigators suggest that nodes larger than 15 mm should be considered abnormal (10). In the current study, we used the extreme value of nodes size. Larger size of nodes (more than 15 mm) might present a higher likelihood of immune response while the smaller nodes (less than 5 mm) might represent non-activated ones. We selected 36 paired enlarged (defined as minimum diameter more than 15 mm in size assessed by node biopsy) and small lymph nodes (defined as maximum diameter less than 5 mm in size) from 36 patients with operable invasive ductal carcinoma, with 12 pairs of luminal-like tumors, 12 HER2-enriched, and 12 TNBC. Patients underwent surgeries at the Department of Breast Surgery, Fudan University Shanghai Cancer Center. All patients were screened for the size of axilla nodes by ultrasound before surgery. During node biopsy, we dissected two nodes (one large and the other small) and incised parts of their medullas for frozen section examination and subsequent RNA extraction if the tumor was proved to be pathologically negative. The remaining part of the two nodes, as well as the remaining nodes obtained by axilla lymph node dissection, were sent to the Department of Pathology. All lymph nodes were pathologically confirmed to be negative. Once any one of the nodes was diagnosed as positive for tumor metastasis, the case was excluded. RNA extraction was performed until the full pathological examination of nodes was finished and the immunohistochemistry results for ER/PR/HER2 were available.

This study was approved by the Institutional Ethics Committee of Fudan University Shanghai Cancer Center. All patients signed informed consent forms.

### RNA Extraction, RNA Sequencing, and Transcriptome Data Analysis

An RNeasy mini kit (Qiagen, Hilden, Germany) was used for purification of total RNA from lymph node tissue. The total RNA samples (1 μg) of extraction of lymph nodes were treated with VAHTS mRNA Capture Beads (Vazyme, Nanjing, China) to enrich polyA+ RNA before constructing the RNA-seq libraries. RNA-seq libraries were prepared using VAHTS mRNA-seq v2 Library Prep Kit for Illumina Xten (Vazyme, Nanjing, China) following the manufacturer’s instructions. Briefly, polyA+ RNA samples (approximately 100 ng) were fragmented and then used for first- and second-strand cDNA synthesis with random hexamer primers. The cDNA fragments were treated with DNA End Repair Kit to repair the ends, then modified with Klenow to add an A at the 3’ end of the DNA fragments, and finally ligated to adapters. Purified dsDNA was subjected to 12 cycles of PCR amplification, and the libraries were sequenced by Illumina sequencing platform on a 150 bp paired-end run. Sequencing reads from RNA-seq data were aligned using the spliced read aligner HISAT2, which was supplied with the Ensembl human genome assembly (Genome Reference Consortium GRCh38) as the reference genome. Gene expression levels were calculated by the FPKM (fragments per kilobase of transcript per million mapped reads). The calculation of abundance of cell types in lymph nodes was conducted using the xCell tool^1^, which can infer the abundance of 64 immune cells and stromal cells based on RNA-seq and microarray data. Gene Set Enrichment Analysis (GSEA) was performed using the GSEA software (v3.0) and the Molecular Signature Database (v7.0). Hierarchical clustering was performed using Euclidean distance as the clustering distance and the average linkage method. The heatmap described the differently expressed coding genes, which were defined as | log2(Fold change, FC)| > 1.0 and *P*-value < 0.05, genes with unknown function (for example, LOC100996401) were also excluded.

### Statistical Analysis

The endpoint for survival analysis was breast cancer-specific survival (BCSS), calculating from the date of diagnosis to the date of breast cancer-specific death. Patients who died of other causes were censored. Age of diagnosis and tumor size were converted into categorical variables. Grade I and grade II patients were merged because of limited numbers of patients, and races of Asian/Pacific Islander and American Indian/Alaska Native were combined for a similar reason. Comparison of clinicopathological characteristics was conducted between patients with cN0 or cN1 disease by χ2 test or Fisher’s exact test if necessary. The Kaplan–Meier method was applied to plot survival curves, with the log-rank test to compare univariate survival differences. A Cox proportional hazard model was used for multivariate analysis and to calculate hazard ratio (HR) with 95% confidence interval (CI). All these statistical analyses were performed using SPSS 23.0 (IBM Corp, Armonk, NY, United States).

Comparisons of xCell scores between large and small lymph node groups were conducted by paired *t*-test using GraphPad Prism 8.0 (GraphPad Software, San Diego, CA, United States). The heatmaps were generated using the MORPHEUS tool (software.broadinstitute.org/morpheus/). Statistical significance was determined with two-sided *P* < 0.05.

## Results

### Baseline Characteristics of Patients

First, we compared the survival of patients with clinically positive nodes (cN1) with that of patients with clinical negative nodes (cN0) in pathologically confirmed node-negative (pN0) TNBC. Theoretically, cN1 had larger and palpable nodes, while cN0 tended to be small and undetectable by imaging tests (according to AJCC staging system). We identified women with pN0 breast cancer disease from the current SEER database. Among them, we compared the cN1 patients of BCSS with cN0 patients. A total of 692 eligible patients were selected from the SEER database, including 359 (51.9%) patients with cN1 and 333 (48.1%) patients with cN0 disease. The median follow-up time was 55 months. The basic information on patients’ clinicopathological variables by cN status in the whole and different subgroups (luminal-like, HER2-enriched, and TNBC) is shown in Table 1.

**TABLE 1:** Baseline characteristics of patients with pN0 breast cancer from SEER database.

Characteristics	Whole (*N* = 692)			TNBC (*N* = 198)			Luminal-like (*N* = 394)			HER2-enriched (*N* = 100)		
	cN0 *N* = 333 (%)	cN1 *N* = 359 (%)	*P*	cN0 *N* = 84 (%)	cN1 *N* = 114 (%)	*P*	cN0 *N* = 210 (%)	cN1 *N* = 184 (%)	*P*	cN0 *N* = 39 (%)	cN1 *N* = 61 (%)	*P*
**Age (years)**												
≤50	132 (39.6)	175 (48.7)	0.016	48 (57.1)	60 (52.6)	0.565	68 (32.4)	87 (47.3)	0.003	16 (41.0)	28 (45.9)	0.632
>50	201 (60.4)	184 (51.3)		36 (42.9)	54 (47.4)		142 (67.6)	97 (52.7)		23 (59.0)	33 (54.1)	
**Race**												
White	242 (72.7)	265 (73.8)	0.856	55 (65.5)	82 (71.9)	0.528	159 (75.7)	139 (75.5)	0.592	28 (71.8)	44 (72.1)	0.153
Black	59 (17.7)	58 (16.2)		21 (25.0)	21 (18.4)		30 (14.3)	31 (16.8)		8 (20.5)	6 (9.8)	
Others^a^	32 (9.6)	36 (10.0)		8 (9.5)	11 (9.6)		21 (10)	14 (7.6)		3 (7.7)	11 (18.0)	
**Grade**												
I-II	146 (43.8)	88 (24.5)	<0.001	13 (15.5)	12 (10.5)	0.387	122 (58.1)	64 (34.8)	<0.001	11 (28.2)	12 (19.7)	0.323
III	187 (56.2)	271 (75.5)		71 (84.5)	102 (89.5)		88 (41.9)	120 (65.2)		28 (71.8)	49 (80.3)	
**Pathological size (cm)**												
≤2	139 (41.7)	72 (20.1)	<0.001	22 (26.2)	24 (21.1)	0.401	105 (50.0)	33 (17.9)	<0.001	12 (30.8)	15 (24.6)	0.497
>2	194 (58.3)	287 (79.9)		62 (73.8)	90 (78.9)		105 (50.0)	151 (82.1)		27 (69.2)	46 (75.4)	
**Adjuvant chemotherapy**												
Yes	237 (71.2)	354 (98.6)	<0.001	72 (85.7)	114 (100.0)	<0.001	130 (61.9)	180 (97.8)	<0.001	35 (89.7)	60 (98.4)	0.074
No/unknown	96 (28.8)	5 (1.4)		12 (14.3)	0 (0.0)		80 (38.1)	4 (2.2)		4 (10.3)	1 (1.6)	

*Abbreviations: cN, clinical lymph node category; pN, pathologic lymph node category; ER, estrogen receptor; PR, progestogen receptor; HER2, human epidermal growth factor receptor-2; and TNBC, triple-negative breast cancer. ^*a*^Others: including Asian/Pacific Islander and American Indian/Alaska Native.*

### Effect of Clinical Node Status on BCSS in Different Subtypes in pN0 Cases

**FIGURE 1:**
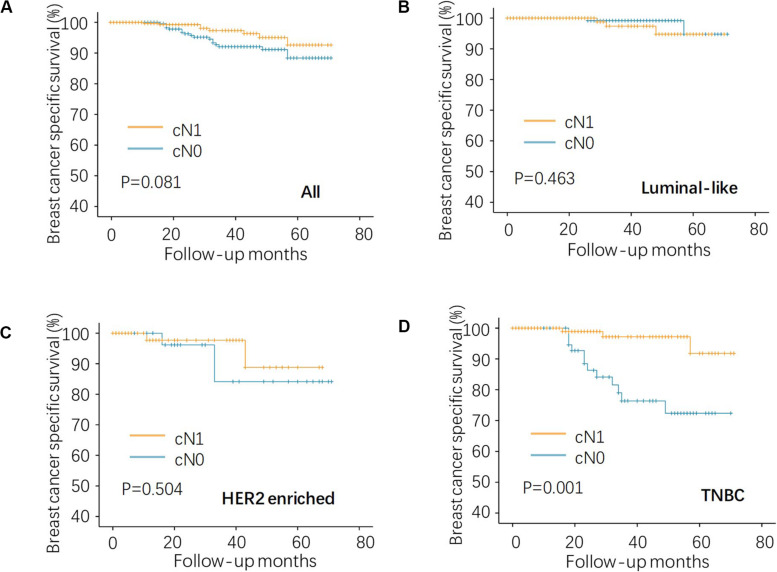
Kaplan–Meier analysis of regional lymph node status on BCSS in the pN0 TNBC. **(A)** the whole, **(B)** luminal-like, **(C)** HER2-enriched, and **(D)** TNBC patients. *BCSS*, breast cancer specific survival; *HER2*, human epidermal growth factor receptor 2; and *TNBC*, triple negative breast cancer.

Given that all the patients had the same pN0 stage, it was expected that there should be no significant difference of BCSS between cN0 and cN1. The results were consistent with this logical expectation in the whole pN0 group (Figure 1A, *P* = 0.081), as well as in the luminal-like subgroup (Figure 1B, *P* = 0.463), and the HER2-enriched subgroup (Figure 1C, *P* = 0.504). Contrary to our expectations, cN0 cases exhibited worse BCSS than cN1 cases in the TNBC subgroup (Figure 1D, *P* = 0.001). Multivariate analysis after adjusting for other confounding factors reconfirmed the findings in TNBC (Table 2, adjusted HR of 0.15, with 95% CI: 0.04–0.55, *P* = 0.004). It is quite anomalous that cN0 had an unexpected worse survival compared with cN1. The potential explanation might be that the cN1 cases in the present study probably had immune-activated lymph nodes with larger size but actually pathologically negative. Based on body examination or imaging tests, physicians might treat larger nodes as metastatic ones and classify them as cN1.

**TABLE 2:** Survival analysis on breast cancer-specific survival among patients with pN0 TNBC in the SEER cohort.

Characteristics	Univariate analysis		Multivariate analysis^a^	
	Hazard ratio (95% CI)	*P*-value	Hazard ratio (95% CI)	*P*-value
**Lymph node status**				
cN0	[reference]	–	[reference]	–
cN1	0.161(0.045–0.571)	0.001	0.148 (0.040–0.546)	0.004
**Age (years)**				
≤50	[reference]	–	[reference]	–
>50	0.914(0.325–2.576)	0.866	0.980 (0.325–2.950)	0.971
**Race**				
White	[reference]	–	[reference]	–
Black	2.139(0.741–6.171)	0.160	1.767 (0.571–5.461)	0.323
Others	1.431(0.179–11.456)	0.736	1.311 (0.153–11.237)	0.805
**Grade**				
I-II	[reference]	–	[reference]	–
III	1.156(0.261–5.123)	0.849	1.573 (0.317–7.815)	0.580
**Pathological size**				
≤2 cm	[reference]	–	[reference]	–
>2 cm	2.375(0.534–10.557)	0.256	3.181 (0.663–15.263)	0.148
**Adjuvant chemotherapy**				
No/unknown	[reference]	–	[reference]	–
Yes	0.679(0.152–3.033)	0.612	0.836 (0.152–4.608)	0.837

*^*a*^Adjusted for lymph node status, age, race, grade, tumor size, and chemotherapy. Abbreviations: cN, clinical lymph node category; pN, pathologic lymph node category; HR, hazard ratio; TNBC, triple-negative breast cancer; and CI, confidence interval.*

### Differentially Expressed Genes in Large and Small Lymph Nodes

**FIGURE 2:**
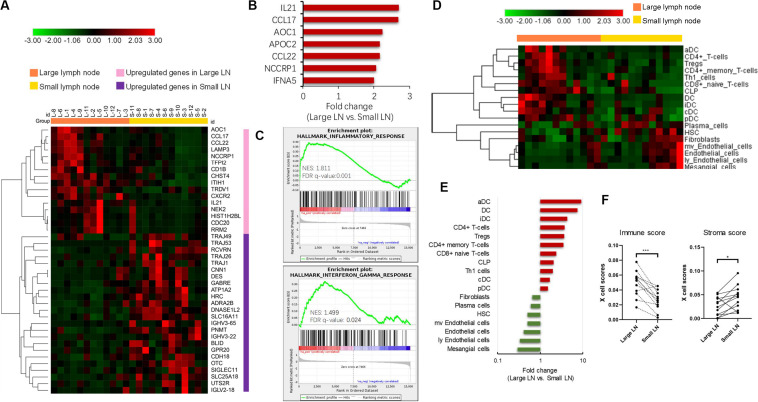
Transcriptome analysis of paired enlarged and small lymph nodes. **(A)** The heatmap of differently expressed coding genes in two groups. **(B)** The most up-regulated genes in enlarged lymph nodes. **(C)** Gene Set Enrichment Analysis (GSEA) analysis of significant activated pathway in enlarged lymph nodes. **(D)** The heatmap of different cell subsets abundance in two groups. **(E)** The fold change of significantly different cellular abundance. **(F)** The comparison of xCell estimated immune and stromal scores in two groups. *P < 0.05; ***P < 0.001.

To investigate the potential molecular events behind enlarged but pathologically negative nodes, we extracted total RNA from 12 paired large (defined as minimum diameter more than 15 mm in size) and small lymph nodes (defined as maximum diameter less than 5 mm in size) from 12 TNBC patients and performed RNA sequencing. Each patient provided 1 pair of 1 large node and 1 small node if available. We chose paired samples from one patient to reduce the interindividual heterogeneity. The heatmap (Figure 2A) described the differentially expressed coding genes. The large node group displayed up-regulation of genes involved in innate and adaptive immune responses compared with the small node group. The heatmap showed in Figure 2A included two types of genes, one related to immune activation and another related to T cell receptor and Ig repertoire. The former class was mainly enriched in enlarged LN and the latter class seemed to express higher in small LN. The most differentially expressed genes are shown in Figure 2B, mainly including immune-related genes such as IL21, CCL17, AOC1, CCL22, and IFNA5. GSEA analysis unveiled the enriched inflammatory and interferon-gamma response pathways in enlarged nodes (Figure 2C). We also performed the same analyses in additional 24 pairs of large and small nodes from 12 luminal-like patients and 12 HER2-enriched patients, respectively. In the HER2-enriched subtype, the results were similar to the findings in TNBC, but the expression intensities of immune-related genes were less than those in TNBC (*P* < 0.05 for IL21, CCL17, and CCL22). The luminal-like subtype seemed to have poor immunogenicity. GSEA analysis did not indicate an adequate and enriched immune-reaction pathway based on the limited differentially expressed immune-related genes in this type (data not shown).

### Differential Immune Cell Abundance in Large and Small Lymph Nodes

We further explored the cellular landscape of large nodes compared to small ones from patients with TNBC using the xCell tool to enumerate cell subsets from transcriptome data. Cell subsets that showed significant difference between two groups (*P*-value < 0.05) and FC > 2 in the heatmap (Figure 2D) were chosen. Large nodes were infiltrated with more immune cell subsets, while small ones were infiltrated with more stromal cell subsets. The FC values of cell subsets are illustrated in Figure 2E by rank, with significantly increased dendritic cells, especially activated dendritic cells, CD4+ T-cells, and CD8+ T-cells. Paired comparisons of immune and stromal scores, which were estimated by cell abundance between the two groups, indicated an up-regulated immune response in large nodes (Figure 2F).

## Discussion

In the current study, we investigated the immunogenomic portrait of clinically enlarged but pathologically negative lymph nodes. Immune activation might be the probable mechanism for the fact that cN1 patients had improved survival compared with cN0 patients in the TNBC subtype.

Lymph node status is one of the most important predictive factors in breast cancer. Clinical assessment for lymph nodes is conducted to provide preoperative information of axilla and aids surgical decision-making. However, clinical examination is not reliable enough due to its limited sensitivity and specificity (11), resulting in the critical need for pathological assessment. The discrepancy that exists between these two kinds of evaluations has been widely reported, and most studies have mainly focused on the false-negative results of clinical evaluation while ignoring the false-positive situation (9, 12). Sacre et al. compared the clinical assessments to pathological findings and found 29% false-positive cases in addition to 45% false-negative cases, implying that the false-positive population deserved equal attention for its notable amount (9). However, much uncertainty still exists regarding the clinicopathological features and survival outcomes of patients with so-called “false-positive” nodes, leading to the purpose of the current study.

Immunotherapy is an evolving therapeutic option with recent encouraging results across multiple tumors (4). However, in breast cancer, a limited response to this novel class of treatment has been seen for poor immunogenicity in breast cancer (13, 14). Subsequently, several studies that focused on the anti-tumor immune response in the setting of molecular stratification uncovered certain immunogenic subtypes of breast cancer. For example, tumor-infiltrating lymphocytes, which provide insight into hosts’ anti-tumor immunity, were present at the highest levels in TNBC with favorable prognosis (15, 16). Identifying those high immunogenic subgroups with clinicopathological biomarkers could help to select potential candidates for immunotherapy in breast cancer. We searched in the SEER database for early-stage breast cancer cases with available clinical and pathological lymph node information and found 359 cN1&pN0 patients. Survival analysis on BCSS showed that cN1&pN0 patients had improved BCSS compared with cN0&pN0 only in the TNBC subtype, which has been considered as the most immunogenic subtype of breast cancer due to its high genomic instability and mutation burden (17). Compared to other breast cancer subtypes, TNBC is the subtype most relative to TILs infiltration and PD-1/PD-L1 expression and has different subcategory related to “hot tumor” or “cold tumor,” which brought clinical predictive and prognostic difference. Considering that infiltrated immune cells present in tumor microenvironment could migrated from lymphoid organs, it was not difficult to understand that heterogeneous immunity might also be seen in regional lymph nodes in TNBC and bring better clinical value than other subtypes. We previously classified TNBC into three heterogeneous clusters involving one “immune inflamed” cluster characterized by the infiltration of adaptive and innate immune cells, suggesting the possibility of administrating immune checkpoint inhibitors in this segment of patients (18).

A potential explanation of our survival results was that the enlargement of nodes represented reactive hyperplasia, which was a regional response of hosts’ systemic anti-tumor immunity. The immune response involves multiple organs and tissues across the body. However, most studies have focused mainly on the local immune response in the tumor or peritumoral microenvironment, regardless of the systematic immune dynamics. Recently, several studies showed their interests in systemic anti-tumor immunity. Spitzer et al. found that patients responded poorly to immunotherapy when the migration of immune cells from the secondary lymphoid organs to tumor environment was suppressed, implying that the immune response was systemic (19). Regional lymph nodes, as the closest lymphoid tissue to a tumor, displayed vital and complex immune responses during tumor regression. The proliferation and activation of lymphocytes in regional nodes, which might appear to be clinically “pseudo-positive” enlarged nodes, actually served as an indicator for the activated immune system. Our study investigated the immunological portrait of enlarged negative lymph nodes compared with small lymph nodes. The results showed that enlarged lymph nodes were infiltrated with more immune cells rather than stromal cells. Further investigation into immune cell types revealed that the total and subcategory of dendritic cells (aDC, iDC, cDC, and pDC) were significantly enriched in enlarged lymph nodes. Additionally, CD4+ T-cells including Th1, Treg, CD4+ memory T-cells also had high abundance in enlarged lymph nodes. However, though up-regulated infiltration of CD8+ naïve T-cells, activated CD8 cells were absent in large lymph nodes, which was consistent with non-differential genes relative to cytotoxic response between large and small lymph node. Thus, the enlarged regional lymph nodes might represent strong ability to present antigen and facilitate the activity of other immune cells when targeting tumor cells in the future. Our study might have clinical implication. The cN1&pN0 status, which presents relatively high immunity and predicts better survival in TNBC, is expected to be paid more attention and serves as convenient evaluation method of immunity to preliminarily select patients suitable for immunotherapy.

Our study had several limitations. First, the case numbers in different subgroups were insufficient for a powerful statistical analysis. Second, though cN2&pN0 were technically excluded, we could not rule out the possibility that there might be a few down-staged cN1&pN0 patients who were administered neoadjuvant chemotherapy in the SEER database. For those patients, the pathologically negative results of lymph nodes may be due to chemotherapy. However, mingling cN2 patients who received neoadjuvant chemotherapy would probably compromise the survival of cN1&pN0 patients, meaning that the survival of cN1&pN0 would be even better if cN2 cases were eliminated.

Taken together, we revealed that clinically enlarged but pathologically negative regional lymph nodes might serve as an indicator for early systemic immune responses to tumors. The elevated expression of immune-related genes and activated immune pathways in regional lymph nodes might confer a survival advantage in TNBC.

## Data Availability Statement

The datasets presented in this study can be found in online repositories. The names of the repository/repositories and accession number(s) can be found below: The NCBI Sequence Read Archive (BioProject: PRJNA658606).

## Ethics Statement

The studies involving human participants were reviewed and approved by Institutional Ethics Committee of Fudan University Shanghai Cancer Center. The patients/participants provided their written informed consent to participate in this study.

## Author Contributions

K-DY and Y-YC designed the study. K-DY, DM, and J-YG collected the data. K-DY, Y-YC, and DM did the statistical analyses. All authors interpreted the results, wrote the manuscript, read and reviewed the manuscript, and approved the final version of the manuscript.

## Conflict of Interest

The authors declare that the research was conducted in the absence of any commercial or financial relationships that could be construed as a potential conflict of interest.

## Footnotes

1 http://xCell.ucsf.edu/

## Abbreviations

AJCC: American Joint Committee on Cancer
BCSS: breast cancer-specific survival
CI: confidence interval
cN: clinical lymph node
ER: receptors: estrogen receptor
GSEA: Gene Set Enrichment Analysis
HER1: Human epidermal growth factor receptor 2
HR: hazard ratio
PD-1: programmed death receptor-1
PD-L1: programmed death receptor ligand-1
pN: pathological lymph node
PR: progestogen receptor
SEER: Surveillance Epidemiology and End Results
TNBC: triple-negative breast cancer.
